# Can leaded glasses protect the eye lens in patients undergoing neck computed tomography?

**Published:** 2021-07-16

**Authors:** Reza Abedi, Naser Ghaemian, Ali Shabestani Monfared, Mohammad Kiapour, Razzagh Abedi-Firouzjah, Fatemeh Niksirat, Alaba Tolulope Agbele, Kourosh Ebrahimnejad Gorji

**Affiliations:** ^1^Student Research Committee, Babol University of Medical Sciences, Babol, Iran; ^2^Department of Radiology and Radiotherapy, School of Medicine, Babol University of Medical Sciences, Babol, Iran; ^3^Cancer Research Center, Health Research Institute, Babol University of Medical Sciences, Babol, Iran; ^4^Department of Medical Physics Radiobiology and Radiation Protection, School of Medicine, Babol University of Medical Sciences, Babol, Iran; ^5^Department of Medical Physics, Tehran University of Medical Sciences, Tehran, Iran

**Keywords:** neck computed tomography, radioprotective glasses, eye lens dose, thermo luminescence dosimeter

## Abstract

**Background and Aims:**

Computed tomography (CT) is one of the main sources using ionizing radiation. Considering the toxicity from this radiation, any technique that could reduce the radiosensitive organs’ doses without affecting the image diagnostic quality must be considered in routine practice. In this study, the amount of eye lens dose reduction in the presence of radioprotective glasses was evaluated in neck CT examinations.

**Methods:**

Thirty adult patients (15 men and 15 women) with a mean age of 44.6 years undergoing neck CT examination participated in this study. For each patient, six thermoluminescent dosimeters (TLDs-100) were attached above the eye lens glasses surface, and another six under the glasses to assess the radioprotective effect of the glasses. The TLDs were readout and converted to Hp (3) as an indicator of eye lens dose. The obtained results from the TLD readouts as eye lens dose were compared using a paired t-test.

**Results:**

The TLD measurements showed the mean±standard deviation values of 2.97±0.61 mGy and 1.04±0.16 mGy for TLDs above and under the radioprotective glasses, respectively. The radioprotective glasses significantly decreased the eye lens dose by about 64.9% (*P*=0.001).

**Conclusions:**

Due to the results, wearing radioprotective glasses for patients during neck CT scans could significantly reduce the eye lens doses.

**Relevance for Patients:**

The outcome of this research shows that leaded glasses can decrease the received dose significantly in patient during neck CT scans.

## 1. Introduction

Computed tomography (CT) is one of the main modalities used in medical imaging and an appropriate choice in the diagnosis of many diseases and health problems [1]. CT examination is one of the main sources of cumulative effective radiation dose [2-4]. In addition, there is an increase in the number of CT examinations due to higher diagnostic value of CT images along with fast image acquisition procedures [5].

Patients’ radiation dose in CT examination is significantly higher than the normal radiographic procedures. For instance, the effective dose in the head and neck region for patients undergoing CT scan is approximately 10 times higher than that of radiographic examination [2]. As a result, concerns about radiation side effects and secondary cancer incidence have been raised for patients undergoing CT examinations compared to other modalities [6,7].

Neck CT scan is one of the primary diagnostic tools for common examinations in evaluating head and neck neoplasms, infections, etc. [8]. Protecting the organs, especially radiosensitive ones, from radiation during the CT examination is very crucial [9]. Furthermore, some of the organs located out of the imaging field may receive scattered and leakage radiation which often leads to an increase in organ dose. Eye lenses are among the radiosensitive organs during neck CT examination. Regarding the International Commission on Radiological Protection (ICRP) reports [10-13], the threshold in eye lens absorbed dose for the induction of deleterious health effect is about 0.5 Gy.

The interaction of ionizing radiation with lens tissue create free radicals and increases the risk of cataract [14,15]. As a result of this, any simple dose reduction technique which does not impair the diagnostic image quality should be taken into consideration. Radiation shielding is a challenging issue regarding the appropriate choice of material and thickness for different energy ranges [16,17].

There are several shielding materials that are designed to ameliorate eye lens doses, these include lead or bismuth shields [17,18], and also flexible shields composed of bismuth in combination with other low atomic number metals [19]. The eyes’ shielding during head or neck CT examinations has been assessed in several studies [18,20]. However, the aforementioned shields are not widely used in routine CT examination due to the hard placement as well as lack of hygienic tips and accessibility. In addition, there are several other techniques for eye lens dose reduction; such as reducing the scan range in routine neck CT [21,22], gantry tilting in brain scan [23], reducing mAs [24], tube current modulation [18], and organ-based tube current modulation (OBTCM) [23,25].

In neck CT examination, the whole or part of the eye may be positioned on the edge of the field of view (FOV) or completely outside the FOV depending on the protocol used in each imaging center, and also the anatomical region which must be illustrated after imaging [21].

Radioprotective glasses, comprised materials equivalent to 0.5–1 mm of lead [20], are designed for protecting eye lens from scattered diagnostic ionizing radiation, but in general, they are not used during patient CT examination [26]. These glasses are relatively low-cost devices that could be found in almost all radiology/imaging centers. In this study, we evaluate the effect of radioprotective glasses in neck CT examination, such that, the eye lenses were completely outside the FOV. Regarding our literature review, no research has assessed the radioprotective effect of these glasses on eye lenses in neck CT examination. The advantage of our method is the simple use of radioprotective glasses compared to lead shields as this could be used for all patients during neck CT scans. Furthermore, radioprotective glasses are much available in imaging centers.

## 2. Materials and Methods

### 2.1. Patients

Thirty adult patients (15 men and 15 women) with a mean age of 44.6 years old (ranging from 21 to 67 years) and with non-emergency situations undergoing neck CT examination participated in this study. All patients were aware of the whole procedures including thermoluminescent dosimeters (TLDs) attached to the radioprotective glasses as well as their eyelid skin. In addition, it was explained to all patients that the eyes must remain closed during the CT procedures. Notably, that the radioprotective glasses are made with a lead equivalent thickness of 0.75 mm, 20 cm^2^-sized, and with a weight of 84 g.

The consent forms were signed by all patients that participated in this study and they know that no invasive procedure would be conducted during their CT imaging. Furthermore, this study was approved by the Ethics Committee and National Research Ethics Board with registration number “IR.MUBABOL.HRL.REC.1398.138”.

### 2.2. Scan protocol

A 16 slice spiral multi-detector Siemens Emotion CT scanner (Siemens Healthcare GmbH, Erlangen, Germany) was used to obtain the CT images of the patients’ necks. All patients were scanned in the supine position with arms lying comfortably beside their body.

The parameters of the neck CT scanning protocols and the mean of the estimated dosimetry parameters of all patients are shown in Table 1. The CARE Dose 4D as well as the automatic exposure control system of the CT scanners was deactivated for all patients to deliver similar exposure parameters. Most of the scans (24 patients) were performed by applying intravascular contrast material (60 mL Iohexol (Omnipaque 300), 2 mL/s, as well as a 35-s scan delay). The scan range was set between external auditory meatus and aortic arch, in such a way that inhibit the eyes from being located in the scan range, which yielded the mean scan length of 23.2±6.9 cm. The reconstructed image matrix in each slice consisted of 512×512 pixels.

**Table 1:** CT scan parameters used for neck examination averaged over all participants

Parameter	Value
Tube voltage (kVp)	130
Effective mAs	80
Rotation time (s)	1
Image slice thickness (mm)	4
Pitch	1
CTDIvol (mGy)	4.8±1.3
DLP (mGy.cm)	172±20
Total scan time (s)	7.8±1.2

### 2.3. TLD location and dose measurement

TLD-100 chips (Harshaw Company, Thermo Electron Corporation, Reading, UK) made of LiF, Mg and Ti with 0.9 mm thickness as well as 3×3 mm^2^ sizes were utilized in this study. All TLDs were calibrated at Iran Secondary Standard Dosimetry Laboratory (ISSDL). Before the measurements began, the dosimeters were annealed in a TLD annealing furnace (1 h at 400°C and 2 h at 100°C) and before the readout, the dosimeters were pre-heated at 100°C for 20 min.

The TLD chips’ calibration process was performed by the following steps: first, the TLDs were exposed using an 80 kVp diagnostic X-ray beam to apply a sensitivity correction, and then, the element correction coefficient (ECC) values were obtained to increase the reproducibility for each TLD. Notably, that a Barracuda dosimeter (RTI Electronics, Sweden) which was calibrated at ISSDL was used for the verification of the dose measurements. Thereafter, the TLDs were irradiated 3 times in free-in-air conditions at the CT scanners’ center of rotation. The ECC for each TLD was calculated using the following equation:

ECC i = TLD i/TLD (mean) (1)

To obtain the calibration factor (CF), the TLDs (ECC close to 1) were exposed 3 times to different selected doses, and the average of TLD readouts was calculated. After this, the dose (mGy) versus TLD reading (nC) was plotted, and the CF was obtained by calculating the slope of the curve.

**Figure 1:**
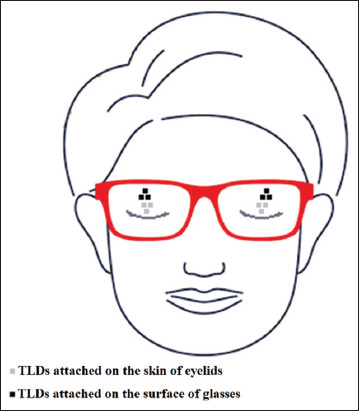
Schematic drawings of the thermoluminescent dosimeters positions under and above the radioprotective glasses undergoing neck computed tomography

During the neck CT examination, the patients wear radioprotective glasses. Six TLDs were attached to the glasses’ surface, and six TLDs were also attached to the patients’ eyelids behind the glasses such that, the radiation attenuation of radioprotective glasses for eye lenses can be assessed (Figure 1).

The Hp (3) was recommended to be used for eye lens dose measurements [12,27]. Following the ICRU Publication 103 [12], for personal dose equivalent, Hp (*d*) is defined as “the dose equivalent in soft tissue as defined in ICRU 51 [28] at an appropriate depth, *d*, below a specified point on the human body”. These represent personal dose equivalent from external exposure in the depth of *d*. We used the reported conversion coefficients from air-kerma to Hp (3) for eye lens dose assessment [29,30]. The surface dose for the eyes was obtained by multiplying the mean values of the TLD readouts, ECC, as well as CF values.

### 2.4. Statistical analysis

The calculated eye lens doses obtained from the TLDs attached to the surface and behind the radioprotective glasses were compared using a paired t-test, performed by SPSS software version 16 (IBM, USA). The significant level was set at P < 0.05. Furthermore, the normality of the data was initially assessed by Kolmogorov–Smirnov statistical test in SPSS software.

## 3. Results

**Figure 2:**
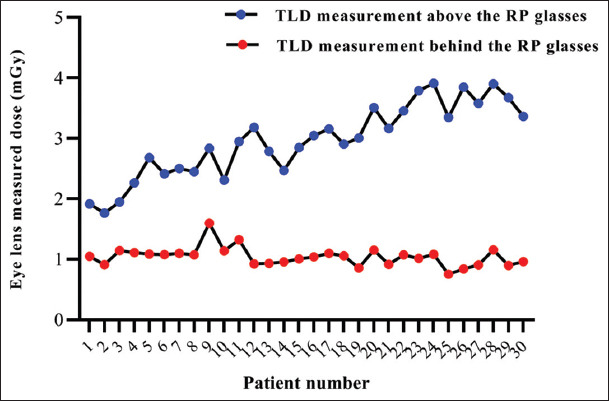
Measured absorbed doses of patients’ eye lenses without (thermoluminescent dosimeters [TLD] measurement on the surface of the RP glasses) and with the RP glasses (TLD measurement behind the RP glasses) for each patient. RP: Radioprotective.

The readout of the TLDs above and behind the radioprotective glasses was converted to Hp (3) doses, these represent the eye lens doses without and with the use of glasses for each patient, have been demonstrated as a chart as shown in Figure 2. The TLD measurements showed the mean±standard deviation values of 2.97±0.61 mGy as well as 1.04±0.16 mGy for TLDs above and behind the radioprotective glasses, respectively. The results of the statistical paired t-test showed that wearing radioprotective glasses significantly decreased the eye lens dose by about 64.9% (*P*=0.001).

## 4. Discussion

Increasing radiation-induced cataract risk with an increase in eye lens dose has been the subject of several studies [15,31-33]. However, due to complications of patients’ follow-up and eye lens dose monitoring, no clear relationship has been established between the eye lens dose and risk of cataract. In a study by Gaudreau *et al*. [31], they reported that one CT examination alone cannot cause cataract or even increase the risk of cataract. On the other hand, ICRP has recommended annual and 5-year cumulative dose limits for eye lenses [12]. Based on the ALARA (as low as reasonably achievable) principle [34], patients’ eye lenses must be prevented from any excess ionizing radiation as much as possible to reduce the radiation toxicities.

Thus, in this present study, we used and evaluated the efficacy of radioprotective glasses in eye lens dose for adult patients undergoing neck CT examinations.

In some of the guidelines from ICRP and National Council on Radiation Protection and measurements, the limits of equivalent dose for eye lenses have been reported at 150 mSv/year [12,35], and both organizations assumed radiation cataract as a deterministic event with a relatively high threshold dose (5–8 Gy). However, the ICRP report 8 noted that the eye may be more sensitive than as considered before. Furthermore, several studies expressed that, the eye lens opacification may occur at much lower exposures (such as 1 Gy) and the development of radiation cataract may be a non-dose threshold event [36-41]. In addition, other factors like individual genetic differences in radiosensitivity could play a key role in explaining the wide variations in the reported time of cataract as well as the degree of opacification [42,43]. In a phantom study by Prins *et al*. [20], they reported that for dental cone-beam CT, radioprotective glasses led to higher eye lens dose reduction in pediatric-phantom. This is because the tissue density of pediatric phantom is less than an adult phantom’s (i.e., the pediatric bone density is lower than the adult bone density). Therefore, there is lower attenuation of the scattered beam, which invariably results in more scattered radiation been absorbed by each organ, thereby causing a higher absorbed dose for children [20].

Considering Figure 2, the protective efficiency of the glasses is different for all patients. This variation could be attributed to different scanning range that was used for each patient and the patients’ size which could also affect the absorption of scattered dose during neck CT examinations.

**Figure 3:**
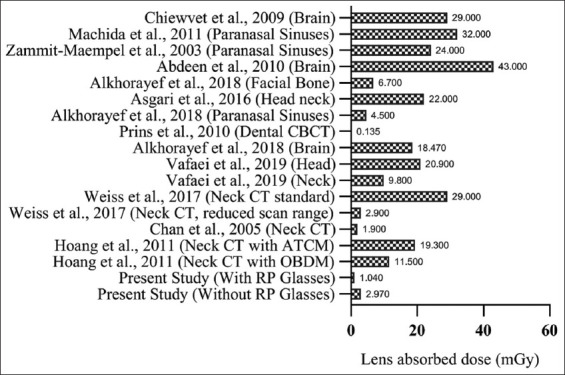
The values of eye lens dose obtained in our study compared to the previous studies during computed tomography scan of head and neck regions. RP: Radioprotective.

Figure 3 shows the values of the eye lens dose obtained in our study compared to previous studies on CT scans of head and neck regions. In this figure, the eye dose values differ from each other mainly due to the scan regions. In some studies, involving head CT or standard neck CT, the eyes are positioned in the scan range, such that, the dose values are relatively high. However, in some other studies like our study, the eyes were excluded from the scan range, such that, the eye lens doses were relatively low. In addition, the exposure and image acquisition parameters can affect the eye lens dose. In a study by Vafaei *et al*. [44], eye lens doses in trauma patients undergoing head and neck CT examinations were measured using TLDs. The eye lens doses for head and neck CT exams were 9.8±3.8 and 20.9±9.6 mSv, respectively. In another study [45], the authors evaluated the eye lens dose during head and neck CT examinations using phantoms and patients. The dose values were in the range of 3.04 to 3.93 mGy for different techniques and situations.

In terms of techniques, several studies have utilized different method. These techniques include; shielding [17,18], reducing the scan range in routine neck CT [21,22], gantry tilting in brain scan [23], reducing mAs [24], tube current modulation [TCM] [18], as well as OBTCM [23,25] for eye lens protection in CT examinations. Some of these techniques, such as TCM considered the eyes to be located inside the FOV of the image. Otherwise, some other methods like the gantry tilting, and scan range reduction, assumed that the eye lenses are not located inside the FOV of the imaging system (e.g., neck or brain CT examinations). On the other hand, shielding and reducing mAs techniques can be used in both cases (i.e., the eye lenses located either inside or outside the FOV) [18].

Although radioprotective glasses are more available than bismuth or other eye lens shields, they are rarely being investigated and based on our knowledge; no study is investigating them in neck CT scans of patients. Furthermore, using TLDs attached above and behind the glasses would enable us to do further eye lens dose monitoring of patients undergoing radiological examinations. In other words, when the lead glasses with the TLDs was placed in the front and at the back of the patient’s head and a CT scan is performed, the readout was different for both TLDs as this represent the dose reduction for the area covered by the lead glasses.

The disadvantage of using radioprotective glasses and bismuth shields compared to tube current modulation technique is that; they cannot be used during imaging of the regions which also include the eyes, due to the metal/high-density artifacts of these shields resulting in low image quality [46]. Recently, new shields, constructed from a combination of bismuth powder with other metals powder having a lower atomic number such as iron and copper, have been developed and can be used during CT examination without any significant effect on image quality of the anatomical regions behind the radiosensitive organs [19]. For instance, in the previous studies [47,48], the authors have declared that bismuth-silicon composite shields and bismuth oxide nanoparticles can reduce breast dose during coronary/chest CT examinations. In addition, Malekzadeh *et al*. [49] expressed that the composites, comprehensively based on a silicon rubber containing various ratios of micro- and nano-barium sulfate (BaSO_4_), lead oxide (PbO), as well as tungsten oxide (WO_3_) particles had significant shielding properties against radioactive sources. However, these shields are not commercially available and their radioprotective ability is under investigation. Furthermore, the glasses used in our study are accessible in almost all medical imaging centers, with ease of use and at a very low-cost.

**Figure 4:**
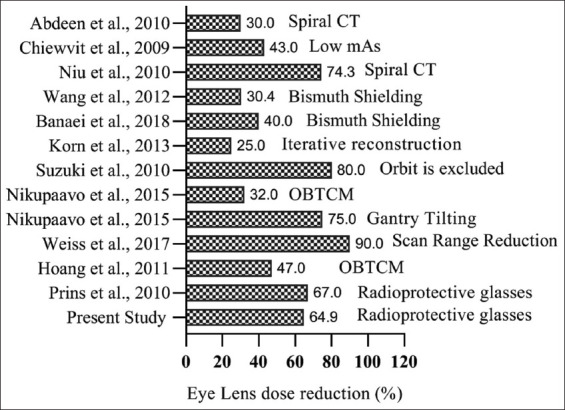
The amount of eye lens dose reduction (%) in our technique compared to other dose reduction methods reported in the previous studies for computed tomography scan of head and neck regions

Figure 4 illustrates the eye lens dose reduction amounts (%) in our technique compared to other methods reported in previous studies for CT scans of head and neck regions. It could be seen that the reduction of scan range has a higher effect on eye lens dose, that is, bringing out the eyes from the primary radiation field must be considered as the best technique to reduce the eye dose. Organ based tube current modulation (OBTCM) and automated tube current modulation (ATCM) were compared in terms of their capability in reducing the delivered dose of some sensitive organs such as eye lens, brain, bone marrow, lung, thyroid, and esophagus in a study conducted by Hoang *et al*. [25]. OBTCM showed a significantly higher radioprotective effect for eye lens compared to ATCM (by about 40%) [25]. However, using OBTCM need more accurate patient positioning in CT scanner and this necessitates more time for patient positioning [19,50]. In addition, OBTCM is not easily accessible in all CT machines, but ATCM could be found in today’s clinical CT machines. The effect of scan range in Z-axis (longitudinal direction) in reducing the eye lens dose alongside diagnostic performance for patients undergoing neck CT scan has been investigated in a study by Weiss *et al*. [21]. They reported that reducing the scan range in Z-axis resulted in eye lens dose reduction from 29.0±11.2 mSv to 2.9±1.8 mSv (*P*<0.001).

Using helical/spiral CT compared to multi-detector sequential CT for the purpose of organ dose reduction has also been evaluated in previous studies but with contradictory results. For example, Toossi *et al*. [51] expressed that spiral head and neck CT exams increased the eye lens dose. In contrast, Chan *et al*. [52] reported that spiral neck CT reduced the eye lens dose from 9.7 mGy to 1.86 mGy (81% reduction). This mismatch may be related to the different settings and image acquisition parameters used. There are also several other studies that reported spiral CT reduces eye lens dose compared to multi-detector sequential CT scans [53,54]. In general, spiral CT scans would be an appropriate option for reducing the eye lens dose with the correct settings of exposure and data acquisition technical parameters.

Although in this present study, we did not evaluate the image reconstruction algorithm effects on the eye lens dose reduction, because some studies have reported the algorithm effects. For instance, in a study by Korn *et al*. [55], it has been expressed that eye lens dose will reduce (about 25%) with an iterative reconstruction algorithm. Furthermore, some algorithms can reduce the artifacts resulting from high-density materials, such as the iterative Metal Artifact Reduction Algorithm [56]. Using these algorithms with high-density shields may result in more appropriate image diagnosis along with organ dose reduction and needs further investigation. In addition, some studies investigated the delivered dose to other organs such as the thyroid, brain, and parotids [20,21,45,51] during neck CT scans, however, we focused on eye lens dose since the glasses will have no radioprotective effect on other organs. Prins *et al*. [20] in a phantom study reported that using radioprotective glasses did not have any effect on brain dose reduction in dental CBCT for both adult and pediatric phantoms.

As a suggestion for future research, the effectiveness of radioprotective glasses on the eye lens dose can be compared with other techniques like bismuth shielding and OBTCM in head and neck CT examinations.

## 5. Conclusion

In this present study, emphasis was placed on ameliorating the eye lens doses putting into consideration the image quality using radioprotective glasses for patients undergoing neck CT examinations. The radioprotective glasses were utilized because of their simple ease of use for all patients, and also for the fact that the protective glasses are relatively cost-effective devices that could be found in almost all radiology/imaging centers. Based on the findings of this research work, wearing radioprotective glasses during neck CT examinations significantly ameliorate the eye lens doses (about 64.9%).
